# Fruit Juice Processing Technologies and Their Impact on the Content of Bioactive Compounds—A Review of Current Approaches

**DOI:** 10.1002/fsn3.72073

**Published:** 2026-06-30

**Authors:** Julia Soja, Dariusz Nowak

**Affiliations:** ^1^ Department of Nutrition and Dietetics, Ludwik Rydygier Collegium Medicum in Bydgoszcz, Faculty of Health Sciences Nicolaus Copernicus University in Toruń Toruń Poland

**Keywords:** antioxidant properties, fruit juices, high‐pressure processing, innovative processing methods, pasteurization, polyphenols, sonication

## Abstract

Fruit juices are valued for their antioxidant content and role in a balanced diet, prompting growing interest in novel, non‐thermal processing technologies that better preserve nutritional and sensory qualities compared to traditional thermal methods. The purpose of this review was to determine the effect of various fruit juice processing methods on the content of bioactive compounds in fruit juices. This work compares thermal methods with emerging innovative methods such as high pressure processing, sonication, pulsed electric fields, cold atmospheric plasma, and high pressure homogenization. A review of the current literature indicates that sonication is a promising technique, enabling effective product preservation while maintaining its nutritional quality. Furthermore, sonication often does not reduce the antioxidant activity of fruit juices; in fact, they tend to have higher levels of polyphenols, anthocyanins, and vitamin C compared to thermal methods. High pressure processing is a method which eliminates preservatives and stabilizers, while effectively preserving the nutritional value. This method maintains the stability of bioactive compounds with antioxidant activity compared to pasteurization. Cold atmospheric plasma and high pressure processing have also demonstrated high potential, combining effective microbial control with substantial preservation of bioactive compounds. This work presents an up‐to‐date overview (2002–2026) of fruit juice processing technologies, emphasizing the importance of selecting appropriate preservation methods to optimize the nutritional quality of the final product. It also outlines future research directions and highlights the challenges associated with implementing innovative technological solutions in the fruit juice industry.

## Graphical abstract

Fruit juices contain a variety of bioactive compounds (mainly polyphenols, anthocyanins, carotenoids, and vitamin C) with antioxidant properties. Traditional thermal processing methods may negatively affect the stability of these compounds, leading to reductions in polyphenol and anthocyanin content, decreased vitamin C levels, and antioxidant activity. Innovative non‐thermal methods offer an effective alternative by enabling better preservation of bioactive compounds while maintaining microbiological safety. Therefore, non‐thermal processing methods are considered superior to conventional thermal preservation in terms of retaining the nutritional value of fruit juices while ensuring effective microbial control.

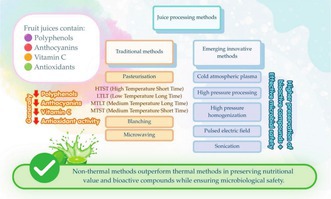

**Abbreviations**
ABTS: 2′‐azino‐di‐(3‐ethylbenzothiazoline)‐6‐sulfonic acid
FRAP: ferric‐reducing antioxidant power
GAE: gallic acid equivalent
HP6P: high pressure processing
HPH: high pressure homogenization
HTLT: high temperature long time pasteurization
HTST: high temperature short time pasteurization
LTLT: low temperature long time pasteurization
MTLT: medium temperature long time pasteurization
PEF: pulsed electric field processing
POD: peroxidase
PPO: polyphenol oxidase
RNS: reactive nitrogen species
ROS: reactive oxygen species
TPC: total polyphenol content
UAE: ultrasound‐assisted extraction
UHT: ultra‐high temperature processing

## 1 Introduction

**FIGURE 1:**
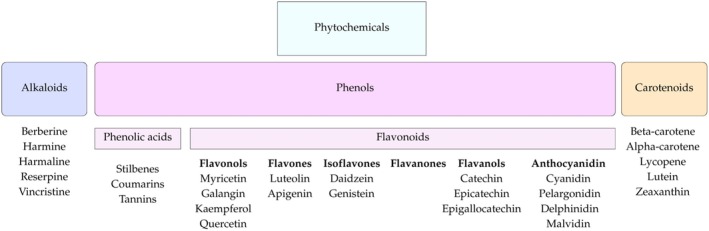
Classification of phytochemicals—modified and based on Nayak et al. (2015), Sumalla‐Cano et al. (2024), Zielińska et al. (2017).

Currently, the growing interest in healthy lifestyles is driving consumers towards products with high nutritional value (Topolska et al. 2021). Despite strong evidence that fruit and vegetable consumption reduces the risk of all‐cause mortality, only a small proportion of the population consumes the recommended five portions per day, and campaigns promoting higher consumption have limited effectiveness (Benton and Young 2019). Fruits are processed into a wide range of intermediate products; however, up to 30% of the fruit mass may be converted into by‐products, including pomace, peels, and seeds. These by‐products also constitute a rich source of bioactive compounds, such as phenolic compounds, peptides, and fatty acids (Patra et al. 2022). Fruit juices are a natural source of vitamins, minerals, and bioactive compounds with antioxidant effects. The detailed classification of phytochemicals is presented in Figure 1.

The antioxidant properties of fruits are attributed to secondary plant metabolites that belong to the group of non‐essential dietary constituents. This means that humans can survive without their dietary intake, no recommended daily intake has been established for these compounds, and their deficiency does not lead to the development of a specific deficiency disease. Nevertheless, the consumption of bioactive compounds through the diet has been shown to effectively contribute to reducing the risk of developing diseases such as cancer, cardiovascular disorders, and diabetes (Patra et al. 2022). Polyphenols, present in fruit in significant amounts, are particularly valued for their antioxidant properties, which may contribute to the reduction of oxidative stress, which has been linked to risk of chronic diseases such as cardiovascular disease (Christiansen et al. 2023, 2022; Habanova et al. 2022; Kasprzak‐Drozd et al. 2021; Parklak et al. 2024; Pokimica et al. 2019), type 2 diabetes (Martiniakova et al. 2025; Zima et al. 2024) or cancer (Gill et al. 2021; Imran et al. 2019; Nowak et al. 2025; Sergiel 2024; Singaravelan and Tollefsbol 2025; Stanca et al. 2024). Juice processing can significantly affect the chemical composition, content of bioactive compounds (including polyphenols) and antioxidant activity of juices (Dubrović et al. 2011). There is a growing body of research in the scientific literature on the effects of different processing methods on fruit juice quality, but the results are often inconclusive, partly due to the wide variety of methodological approaches, including combinations of processing techniques and differences in process parameters such as temperature, pressure, and treatment intensity and depend on the type of fruit and the specific technologies used. Traditional methods, such as pasteurization, can lead to a reduction in polyphenol content and a decrease in antioxidant activity as a result of high temperature (Ma et al. 2013; Vieira et al. 2018). Carotenoids (Atencio et al. 2022; Moura et al. 2023; Souza et al. 2023) and vitamin C (El‐Ishaq and Obirinakem 2015; Iversen 1999; Mieszczakowska‐Frąc et al. 2021) are sensitive to processing temperature, which also determine the antioxidant properties of juices. Carotenoid degradation in juices occurs primarily through isomerization and oxidation mechanisms. The structure of carotenoids is inherently sensitive to heat, light, and oxygen (Dutta et al. 2011). The major degradation pathways are isomerization of trans‐carotenoids to cis‐carotenoids, promoted by acids, heat, and light, reducing color and vitamin A activity and enzymatic and non‐enzymatic oxidation (Kravets et al. 2024). Modern processing methods, on the other hand, allow better preservation of bioactive components (Bhattacharjee et al. 2019).

The antioxidant profile of fruit juices is complex and results from the unique combination of their constituents and their interactions (Durazzo et al. 2019). Examples of compounds with antioxidant activity include polyphenols, flavonoids, carotenoids (including lycopene), and vitamin C (Nayak et al. 2015). Antioxidant compounds play a key role in maintaining the body's homeostasis and protecting cells from free radical damage. Free radicals, such as reactive oxygen species (ROS) and reactive nitrogen species (RNS) (Sies et al. 2017), are formed as natural products of metabolism, while their excess can lead to a specific disruption of homeostasis, such as oxidative stress, which contributes to the development of many chronic diseases such as cancer (Lin et al. 2025), cardiovascular diseases (Berisha et al. 2025; Wan et al. 2024), diabetes (Caturano et al. 2023), or neurodegenerative diseases (Chen et al. 2012; Singh et al. 2019). For this reason, there is a need to search for methods that will reduce the loss of bioactive compounds in fruit juices, while maintaining their microbiological quality.

The aim of this review is to comprehensively analyze and critically evaluate the available scientific studies that investigate how various processing methods—such as thermal treatments and novel non‐thermal technologies—affect primarily the content and stability of bioactive compounds in fruit juices. The review provides information on the benefits and limitations of the various technologies in the context of producing juices with a high nutritional value (including primarily antioxidant components) and identifies research gaps in the current literature on the subject based on the experimental work published to date.

## 2 Methodology

**FIGURE 2:**
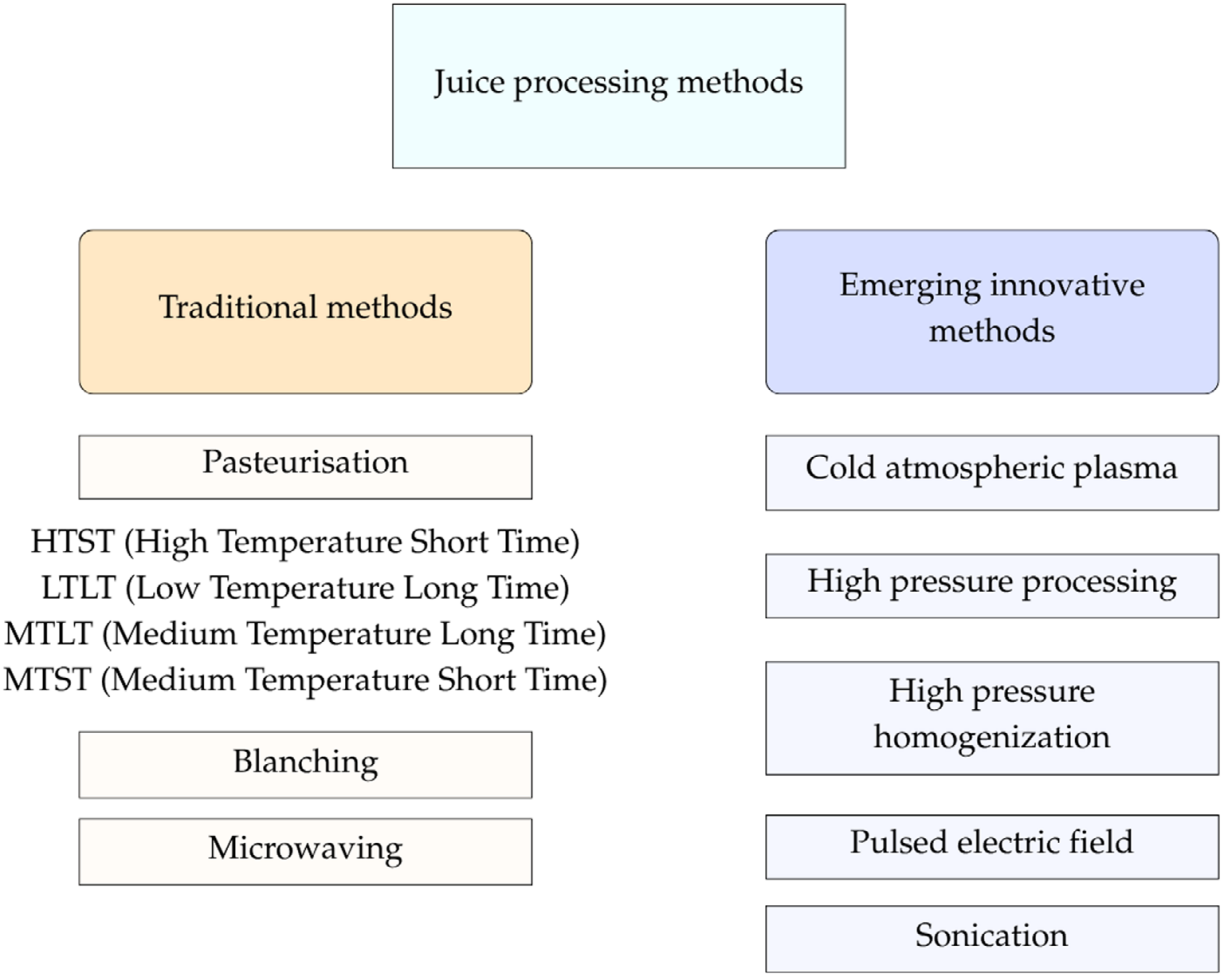
Classification of juice processing methods: traditional and innovative.

This review was conducted according to the principles of narrative literature review. A comprehensive search of the Google Scholar, PubMed and Web of Science databases was performed up to February 2026. The search strategy combined free‐text words related to “fruit juice processing,” “blanching,” “microwaving,” “sonication,” “ultrasounds,” “antioxidant capacity,” “high‐pressure processing,” “cold plasma,” “pulsed electric field,” “polyphenols,” “vitamin C.” The bibliographic entries were selected on the basis of an individual assessment of the title and abstract of the literature searched—papers were included that covered the content of the description of an experimental study on juice or fruit juices (> 80%) (in exceptional cases, experiments on vegetable juices (< 10%) and other derivatives of these two product groups were also included to show the important context of the phenomena described), which were subjected to selected preservation methods to extend the shelf life of the product. The classification of juice processing methods, including both traditional and innovative techniques, is presented in Figure 2.

### 2.1 Pasteurization

Pasteurization is a commonly used heat treatment method in the food industry, which involves heating products to a specific temperature for a set period of time in order to eliminate or reduce pathogens and microorganisms. It allows for a significant increase in the shelf life of fruit juices and other food products without the use of chemical preservatives (Polak et al. 2024). With regard to fruit juices, pasteurization not only plays the role of protecting the product from microorganisms, but also affects the chemical and organoleptic properties of the finished product (Polak et al. 2024; Vieira et al. 2018). Nevertheless, despite its numerous benefits, heat treatment can lead to the degradation of thermolabile bioactive components such as vitamin C, anthocyanins, or some polyphenols (Igual et al. 2010; Mohideen et al. 2015; Petruzzi et al. 2017).

High‐temperature processing methods are widely used in the fruit juice industry due to their effectiveness in extending product shelf life (Nadeem et al. 2022; Petruzzi et al. 2017). The use of high temperature allows the destruction of microorganisms and the deactivation of enzymes responsible for the spoilage of juices and, as a result, significantly extends their shelf life (Nadeem et al. 2022; Pérez‐Lamela et al. 2021; Petruzzi et al. 2017; Rawson et al. 2011). Despite these advantages, these processes can have a significant impact on the chemical composition of juices, including the content of bioactive substances such as vitamins and polyphenols, and consequently on their antioxidant activity (Al‐Juhaimi et al. 2018; Igual et al. 2010; Ma et al. 2013; Petruzzi et al. 2017; Rawson et al. 2011).

Thermal processes can be classified according to the intensity of the heat treatment (Petruzzi et al. 2017). A distinction is made between low‐temperature, long‐term (LTLT) and high‐temperature, short‐term (HTST) (Mirondo and Barringer 2015; Petruzzi et al. 2017) and long‐term, high‐temperature (HTLT) pasteurization processes. The definitions of HTST pasteurization vary among authors, which may complicate direct comparisons between studies. In recent years, there has been an increased interest in medium‐temperature, long‐lasting (MTLT) thermal processing, in which the temperature is less than 80°C and the holding time is more than 30 s. Its advantage is minimal product processing. The last method mentioned is medium‐temperature, short‐term treatment (MTST). It involves a temperature below 80°C and a holding time of less than or equal to 30 s.

The HTLT method, depending on the temperature used, can be classified as pasteurization (less than 100°C), conservation/preservation (approximately 100°C), or sterilization (more than 100°C). The temperature is greater than or equal to 80°C and the heating duration is longer than 30 s (Petruzzi et al. 2017). HTST pasteurization, on the other hand, is the standard method used to pasteurize fruit juices (Mirondo and Barringer 2015). The temperature is approximately 72°C and is maintained for 15 s or more. For different fruit juices, the HTST pasteurization temperature varies from 72°C to less than 100°C, while the treatment time is always 1 min or less (Chen et al. 2013). Slightly different parameters for this pasteurization are reported by Petruzzi et al. (2017) and Ağçam et al. (2018) ‐ a temperature greater than or equal to 80°C and a time less than or equal to 30 s. It was observed that the application of HTST heat treatment improves the color of pomegranate juice. On the other hand, HTST pasteurization can reduce the citric acid and ascorbic acid content of grapefruit juice and the ascorbic acid content of pomegranate juice (Petruzzi et al. 2017). In contrast, the essence of low‐temperature long‐time (LTLT) pasteurization is heating food at about 63°C for at least 30 min (Chen et al. 2013). Alternatively, according to Petruzzi et al. (2017), LTLT pasteurization involves a temperature below 80°C and a holding time above 30 s. This method is not preferred for the pasteurization of fruit juices. It causes problems in obtaining adequate juice quality and, more specifically, in retaining nutrients and flavor. However, LTLT pasteurization is the most economical and can be affordable for small enterprises (Mirondo and Barringer 2015).

Gil‐Izquierdo et al. (2002) evaluated how different orange juice processing methods affect the phenolic compounds, vitamin C content, and antioxidant capacity. The selected methods included squeezing, mild pasteurization (70°C for 30 s), standard pasteurization (95°C for 30 s), concentration (concentration), and freezing. In addition, the use of manual and commercial extrusion was a differentiating factor. The authors concluded that the antioxidant activity of orange juice is mainly due to the high content of ascorbic acid, which provides at least 77% of the total antioxidant capacity and remains stable regardless of the chosen processing technique. It follows that processing had a negligible effect on the antioxidant properties of orange juice due to the stability of ascorbic acid. The opposite result was obtained in a study by Ma et al. (2013). Pasteurization had a negative effect on carrot juice—it reduced total polyphenol content (TPC) and antioxidant activity, including the ability to scavenge DPPH free radicals and chelate Fe^2+^ ions. As a result, pasteurization reduced the antioxidant properties of the juice compared to fresh juice and other treatments (blanching and enzymatic liquefaction). Similarly, a study by Dubrović et al. (2011) showed that heat treatment of strawberry juice at 85°C for 2 min resulted in a 5.3%–5.8% decrease in anthocyanin content compared to untreated juices. This result is consistent with the well‐established observation that anthocyanins are among the most thermolabile phenolic compounds.

In another study, pasteurization of pomegranate juice was compared with a non‐thermal method—pulsed electric field (PEF) (see Section 2.4 for details), and the effect on the content of phenolic compounds, anthocyanins, and antioxidant activity was assessed (Guo et al. 2014).

**FIGURE 3:**
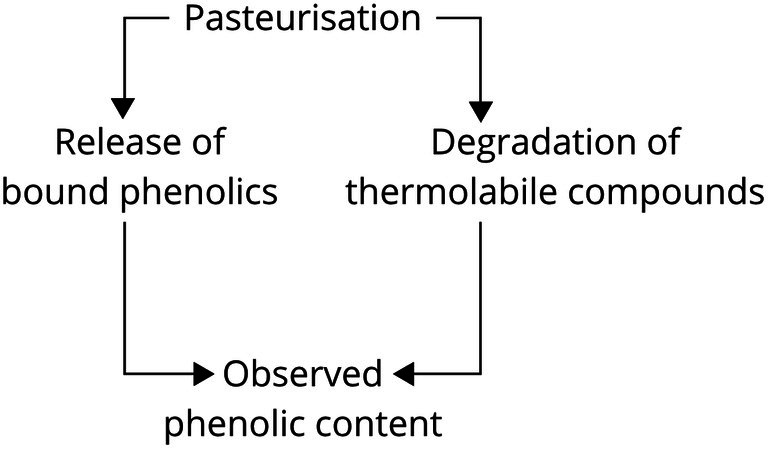
Effect of heat treatment on the plant matrix.

PEF processing had no significant effect on the content of phenolic compounds and anthocyanins compared to fresh juice, while the amount of phenols increased in the heat‐treated sample. The authors explain this by the breakdown of phenolic polymers. This contrasts with studies reporting reductions in total polyphenol content after pasteurization, suggesting that the net effect of heat treatment depends on the balance between degradation of heat‐sensitive phenolics and the release of previously bound phenolic compounds from the plant matrix (Figure 3). The antioxidant activity of the PEF‐treated juice was similar to the untreated juice for the first 4 weeks and did not change significantly during the subsequent storage period. Although the anthocyanin content of all samples decreased over time, the least loss was observed in PEF‐treated juices.

In some pomegranate fruit juices (depending on the variety), a slight increase in anthocyanin content was observed after pasteurization and 2 weeks of storage, which could be due to thermal extraction of previously bound or polymerized anthocyanin molecules and the arrest of their enzymatic degradation (Alighourchi et al. 2008). However, this finding differs from studies reporting anthocyanin losses after thermal treatment, indicating that the response of anthocyanins to pasteurization is highly dependent on fruit type, anthocyanin composition and storage conditions. Our proposed explanation for this phenomenon is that pasteurization inactivates enzymes such as polyphenol oxidase and peroxidase. These enzymes are responsible for the oxidation of phenolic compounds and anthocyanins. Therefore, short‐term thermal treatment may paradoxically increase the stability of certain bioactive compounds during subsequent storage. This may explain the increase in anthocyanin content observed by Alighourchi et al. (2008) after 2 weeks of storage. Studies frequently report the “total anthocyanin content” without considering the qualitative profile of individual anthocyanins. However, polyphenols, as a diverse group of compounds, exhibit different levels of thermal stability. Evidence shows polyphenols generally degrade 15%–30% after 4 h at 60°C–100°C (Volf et al. 2014). However, at 100°C, catechin content declined 15%–96% depending on pH conditions (Zeng et al. 2017). Most polyphenol families remain stable up to 100°C, but significant degradation of epicatechin, resveratrol, and myricetin occurs at 125°C (Liazid et al. 2007). At higher processing temperatures (93°C–135°C), degradation rates vary dramatically by compound type (Human et al. 2021). Evidence spans multiple polyphenol families across diverse experimental conditions, though specific compound‐by‐compound comparisons remain limited. These findings suggest that two juices with similar total anthocyanin contents may respond very differently to the same temperature treatment.

Thermal pasteurization can also affect the concentration of vitamin C in various fruit juices. In a study by Jia et al. (2022), the effects of thermal pasteurization, thermal‐assisted high hydrostatic pressure, and high hydrostatic pressure on the quality of aronia juice were evaluated in this study. The results showed that all methods significantly decreased the aerobic plate counts of aronia juice. Pasteurization had a more negative effect on the color and antioxidant properties of the juice compared to high‐pressure methods. Mandha et al. (2023) propose an explanation that the darkening of juice may be due to Maillard reactions. However, it is noteworthy that mild thermal treatments (50°C–70°C) combined with natural antimicrobial agents such as carvacrol allow less vitamin C loss compared to high‐temperature pasteurization (> 70°C) (Tchuenchieu et al. 2018).

Future research should include the identification of optimal pasteurization parameters. As a noteworthy alternative to pasteurization, ultraviolet (UV) treatment of fruit matrices has been mentioned in literature (Jafari et al. 2026). This approach allows for better preservation of bioactive compounds, does not deteriorate juice quality, and extends shelf life, although to a lesser extent than thermal processing. However, its effectiveness may be reduced in cloudy juices due to the limited penetration of UV light. There is a need to further define optimal pasteurization temperatures and times for different types of fruit juices in order to minimize the loss of bioactive components (e.g., vitamin C, anthocyanins, polyphenols) while maintaining microbiological safety and sensory stability of the products. Studies should take into account the specificities of different fruit varieties, their chemical composition and reactions to heat treatment in order to adapt pasteurization parameters to the characteristics of the raw material. The most significant methodological limitation of the reviewed literature is the lack of consistent definitions of thermal processing conditions. According to some authors, HTST refers to treatment at 72°C for 15 s, whereas others define it as temperatures ≥ 80°C applied for ≤ 30 s. The difference between 72°C and 90°C may result in entirely different chemical transformations. Consequently, studies classified as HTST are often not directly comparable. Moreover, most studies describe changes in the concentrations of bioactive compounds but do not provide a detailed explanation of the underlying mechanisms responsible for their degradation or release. Therefore, future research should focus not only on the quantitative determination of bioactive compounds, but also on the mechanisms of their transformations, their bioavailability, and the changes occurring during product storage.

### 2.2 Blanching

Blanching as a process aims to inactivate enzymes that cause quality deterioration (especially browning, off‐flavors, and nutrient loss). Enzymes inactivated during blanching include peroxidase (POD) and polyphenol oxidase (PPO) (Jabbar et al. 2014; Noreña and Rigon 2018). It allows the final color of the product to be fixed and the nutritional values to be preserved. The most common practices include the use of hot water, steam (also under vacuum conditions), and hot air. Water blanching, for example, can take place at 75°C–95°C for 1–10 min (Ma et al. 2013; Rawson et al. 2011). Ma et al. (2013) reported the preservation of high total polyphenol content (TPC) values in a sample of carrot juice that was blanched at 86°C for 10 min before squeezing (along with enzymatic fixation compared to pasteurization of carrot juice). This process assisted the solubilization of polyphenols in the juice, which translated into an increase in their concentration and antioxidant activity. The differences in polyphenol content between blanching and pasteurization were probably due to the different temperatures and duration of these processes.

**FIGURE 4:**
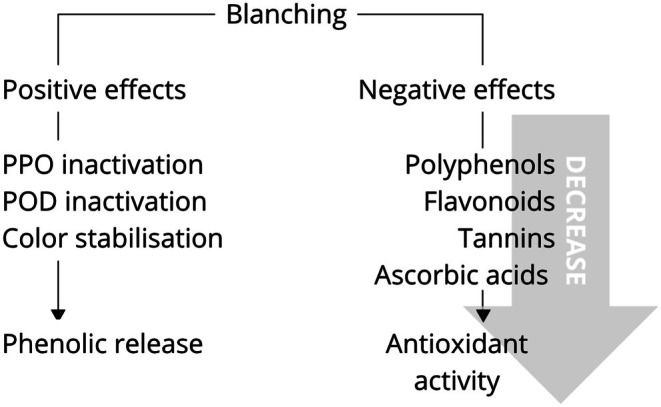
Positive and negative effects of blanching.

Jabbar et al. (2014) blanched carrots in both regular and acidified water (100°C for 4 min), followed by juice extraction and sonication (15°C for 2 min, 70% amplitude, 20 kHz). Blanching effectively inactivated peroxidase; however, it simultaneously reduced the content of bioactive compounds (polyphenols, flavonoids, tannins, ascorbic acid) and antioxidant activity. This method also influenced the acidity of the final product by lowering its pH (Jabbar et al. 2014). It means that although blanching protects plant products from further enzymatic degradation through the inactivation of enzymes such as polyphenol oxidase and peroxidase, it may simultaneously promote the loss of water‐soluble bioactive compounds due to leaching and thermal degradation. The positive and negative effects of blanching are shown in Figure 4.

In recent years, the combined effect of fruit blanching (specifically strawberries) and high‐pressure processing (HPP) has also been evaluated for smoothie production. The combination of blanching and HPP significantly improved the quality of strawberry smoothies compared to traditional thermal processing and untreated samples (Del Carmen Razola‐Díaz et al. 2024).

Despite these promising findings, there remains a lack of comprehensive studies on the application of blanching in fruit juice production. Existing research has primarily focused on vegetable juices (e.g., carrot juice) or more complex fruit‐based products such as smoothies. Although smoothies are fruit‐based, they differ significantly from juices in composition—particularly in fiber content and viscosity—which may influence the effectiveness and mechanisms of processing techniques. Therefore, findings from studies on smoothies or vegetable juices cannot be directly extrapolated to fruit juices. In summary, there is a clear research gap concerning the use of blanching in fruit juice processing. Moreover, contradictory findings have been reported in the literature, as other studies observed reductions in polyphenol content following blanching. This suggests that the final effect depends on the balance between enhanced release of phenolic compounds from plant tissues and their thermal degradation or leaching during processing.

Most available research focuses on synergistic combinations, making it difficult to clearly determine the standalone impact of blanching on the properties of fruit juices, including bioactive compound content, color stability, antioxidant activity, and sensory parameters. However, such data are essential for optimizing processing methods and making informed technological decisions based on reliable and comparable results. Therefore, further research is needed to investigate blanching as an isolated treatment in the context of fruit juice production.

### 2.3 Microwaving

Microwave technology, based on dielectric heating caused by electromagnetic radiation, is widely used in food processing, especially of juices, reducing microbial activity (including *Escherichia coli* ) and reducing the browning of apple juice (Zhang and Zhang 2014). Microwaves exhibit high potential for microbial inactivation due to thermal effects. However, their effectiveness depends on several factors, including power, frequency, and temperature distribution within the product (Kernou et al. 2022). Therefore, microwave heating, by rapidly and evenly heating the entire volume of fruit juice, allows for shorter thermal processing times, minimizing losses of nutrients and sensory quality (Saikia et al. 2016).

**FIGURE 5:**
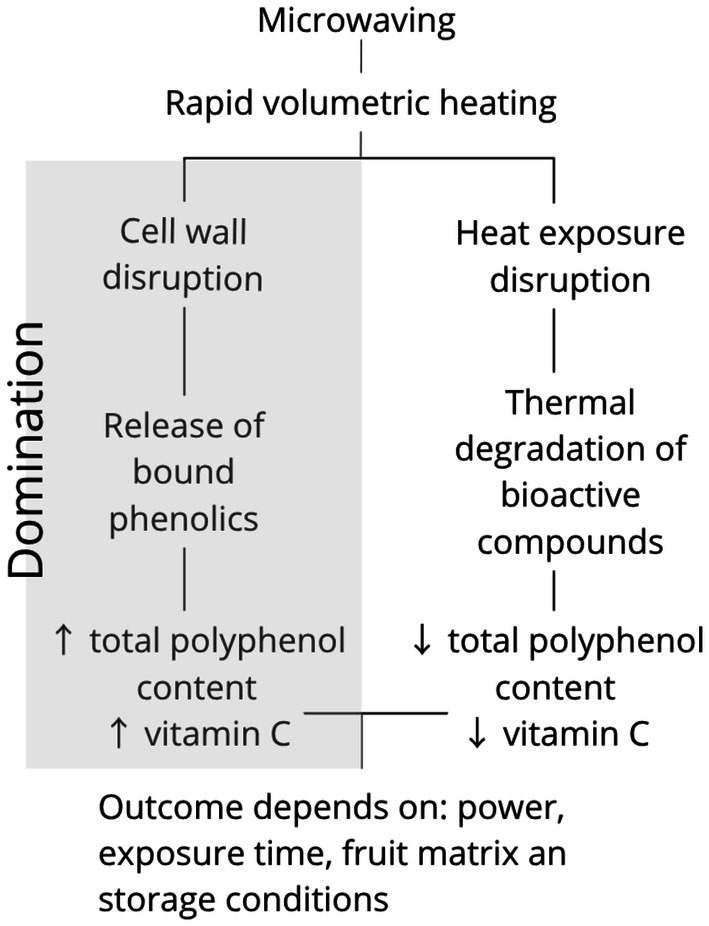
Mechanisms of microwaving fruit juice and bioactive compounds release.

Microwave heating methods have varying effects on antioxidant compounds in fruit juices. In apple juice, microwave treatment (720 W and 900 W for 100 s) increased the total flavonoid and polyphenol content in the sample, thereby increasing the overall antioxidant activity (Zhang and Zhang 2014). Similarly, microwave treatment had a positive effect on the phenolic compound content and antioxidant activity in other fruit juices such as carambola, black yamun, and watermelon (Saikia et al. 2016). This suggests that appropriate selection of microwave treatment parameters may promote the release of phenolic compounds with high biological activity. The literature also discusses the application of the microwave method on other fruit‐derived raw materials. Microwave heating of apple and beetroot waste significantly increased the amount of water‐soluble antioxidants—vitamin C content increased 1.32–1.57 times and flavonoids 1.77–2.01 times (Perfilova et al. 2020). The observed increase in phenolic content may be attributed to the release of compounds previously bound to the plant matrix, the breakdown of phenolic complexes, or enhanced extractability and analytical detectability following microwave treatment. “Matrix” refers to the entire physicochemical system of the juice, including water, sugars, organic acids, soluble dietary fiber, proteins, pectins, polyphenols, and the cellular microstructure. The increase in vitamin C content may be explained by enhanced release of ascorbic acid from plant tissues as well. In citrus peels, moderate microwave power (250 W) and heating time (10 min) improved antioxidant potential by increasing free phenolic fractions while decreasing bound fractions (Hayat et al. 2020). This suggests that appropriate selection of microwave treatment parameters may promote the release of phenolic compounds with high biological activity. The mechanisms of release of bioactive compounds from fruit juice are shown in Figure 5.

The studies cited above indicate that the effect of heat treatment on the chemical composition and antioxidant properties of fruit juices depends on the temperature used, the processing time, and the specific nature of the raw material. Alternative processing methods, such as microwave treatment, may reduce the loss of valuable bioactive components. Some studies even indicate the possibility of an increase in phenolic content after heat treatment, which may be due to the breakdown of phenolic polymers and an increase in the availability of certain compounds. However, this effect is dependent on the type of juice, the treatment method, and the storage time. Pasteurization and heat‐treatment methods remain key technologies in fruit juice production, but their impact on the bioactive content and antioxidant properties of the final product requires further research. Optimal processing parameters need to be developed to minimize nutrient losses while maintaining the microbiological safety and sensory stability of juices. On the other hand, many food ingredients are heat sensitive and can be lost during heat processing. Additionally, rising consumer awareness has increased demands for fresh, higher quality, and microbiologically safer and stable foods and has promoted research on non‐thermal methods of food preservation (Chavan et al. 2022). Although microwave processing is generally considered a thermal technology, its shorter treatment times may reduce quality deterioration compared with conventional heating. Nevertheless, growing consumer demand has also stimulated research into truly non‐thermal preservation methods.

### 2.4 Emerging Innovative Methods

Traditional pasteurization, although effective in eliminating microorganisms, often leads to the degradation of valuable bioactive compounds (further details are provided in the previous section). As a result, there is growing interest in alternative technologies that enable products to maintain quality while extending their shelf life. Modern methods include sonication, cold atmospheric plasma (CAP), high pressure processing (HPP), high pressure homogenization (HPH), and pulsed electric field (PEF).

One modern and promising method for fruit juice processing is sonication, which is the use of high‐frequency ultrasonic waves (20 kHz to 100 kHz) (Chavan et al. 2022) to preserve fruit juice (Bhattacharjee et al. 2019; Nadeem et al. 2022; Rawson et al. 2011). Sonication, due to its ability to inactivate microorganisms at low temperatures, allows for a significant increase in the shelf life of juices without the need for intensive heat treatment (Bhattacharjee et al. 2019; Rawson et al. 2011). This process is based on physical phenomena such as acoustic cavitation, which is created by the action of ultrasonic waves on a liquid medium. Due to fluctuations in acoustic pressure, the bubbles formed in the fluid rapidly grow and collapse (Bhattacharjee et al. 2019; Chavan et al. 2022; Yildiz and Feng 2019).

Ultrasound affects microorganisms through multiple mechanisms, leading to their effective inactivation by combining physical, chemical, and mechanical effects; however, achieving full microbiological safety often requires combining sonication with other preservation methods (e.g., thermal treatments) (Kernou et al. 2022). Studies have shown that sonication effectively reduces pathogenic microflora in fruit juices (Bhattacharjee et al. 2019).

Yildiz and Feng (2019) conducted cherry juice sonication at 20 kHz and 100% amplitude for 2, 3, 5, and 10 min. The longer the juice has been sonicated, the higher levels of total polyphenols, antioxidant activity, and ascorbic acid were recorded. For this reason, the authors suggest the application of 10‐min sonication of fruit juices in the commercial sector. Another study showed that apple juice treated with ultrasound in a water bath had higher antioxidant capacity, while ultrasound had no effect on pH, juice acidity, or dry matter content (Belgheisi and Kenari 2019).

In comparison, in traditional juice production, a large portion of antioxidants remains in the waste (especially in the peels). The study by Qadeer et al. (2025) shows that peels contain more polyphenols than the pulp, and the use of modern extraction methods such as ultrasound‐assisted extraction (UAE) enables the preservation of these compounds. This means that we observe a universal mechanism of UAE action across different matrices.

Nadeem et al. (2022) showed that the application of ultrasound significantly increased the total phenolic content of juices from selected citrus fruits (Chinese orange *Citrus sinensis* , grapefruit *Citrus paradisi*, mandarin *Citrus reticulata* ). The total phenolic content of the juices of all citrus varieties ranged from 223.49 ± 4.5 to 590.47 ± 5.5 μg GAE/g and increased from 315.18 ± 6.1 to 645.44 ± 7.1 μg GAE/g after ultrasonic fixation. DPPH values of juices of all citrus varieties ranged from 791.54 to 1251.93 μmol Trolox equivalent/mL and increased from 1001.54 to 1336.77 μmol Tx/mL after ultrasound fixation. The increase in values was attributed to the release of phenolic compounds due to cell wall breakdown.

Also in a study by Aadil et al. (2013) juice preservation with ultrasound showed beneficial effects on the vitamin C content, phenolic compounds, and antioxidant activity of grapefruit juices. The authors suggested that sonication increases vitamin C content, which is attributed to the removal of oxygen through the cavitation phenomenon (Aadil et al. 2013; Rawson et al. 2011).

The team of Saikia et al. (2016) investigated the effects of ultrasound application on juices from 5 fruits: carambola ( *Averrhoa carambola* L.), black jamun (*Syzygium cumuni* L. Skeels.), watermelon ( *Citrullus lanatus var. lanatus* ), pineapple ( *Ananas comosus* L. Merr) and lychee ( *Litchi chinensis* Sonn.). The effects of different processing methods on the phenolic compound content and antioxidant activity of fruit juices varied. In carambola and lychee juices, sonication increased the total phenolic compound content, while in black yamun juice, the best results were achieved after pasteurization. In pineapple and watermelon juices, the total phenolic compound content decreased after most methods, except for some microwave variants.

In the case of black mulberry juice, ultrasound‐fixed samples showed higher TPC, anthocyanin content, and higher antioxidant activity compared to microwave‐ and heat‐treated juice (Jiang et al. 2015). Similarly, a study by Dinçer and Topuz (2015) observed a slight increase in total anthocyanin content (from 2.4% to 4.1%) in black mulberry juice after ultrasonic fixation.

Mohideen et al. (2015) studied highbush blueberry ( *Vaccinium corymbosum* ) juice, which they sonicated. The study showed that ultrasound can be an alternative preservation method compared to pasteurization. Sonication preserved the anthocyanins and color of the berry juice—the total anthocyanin content (TAC) of the untreated berry juice and the ultrasound‐treated juice were not statistically significantly different (*p* ≤ 0.05). TPC in sonicated juices increased significantly with increased flow rate and amplitude (*p* ≤ 0.05). The highest TPC content was obtained at a flow rate of 93.5 mL/min and amplitude of 100%. Interestingly, it was noted that all ultrasound‐treated juices showed higher TPC content than untreated juice. The authors suggest that this is due to the extraction of some phenolic acids from suspended berry juice particles by ultrasonic treatment.

Dubrović et al. (2011) compared several methods of strawberry juice preservation in their study. After ultrasound treatment (20°C for 3, 6 or 9 min), anthocyanin degradation was attenuated and amounted to 0.7%–4.4% compared to untreated juices. Only in the case of sonication at 55°C and a treatment time of 9 min did the total anthocyanin content, compared to the untreated juice, decrease by 5.8%–7.1%. This suggests that high temperature combined with ultrasound may lead to greater degradation of these compounds. A similar effect was reported by Rawson et al. (2011) using watermelon juice as an example.

A study by Alves Filho et al. (2018) analyzed the effect of thermal (pasteurization HTST and ultra‐high temperature processing (UHT) sterilization) and non‐thermal (ultrasound and plasma) on the composition of acerola juice, with a particular focus on the content of vitamin C and other organic compounds. The authors concluded that both non‐thermal treatments had a significant effect on acerola juice. Whereas, thermal processing HTST and UHT increased vitamin C concentration in pure juice and in juice with inulin. In contrast, in juices with gluco‐oligosaccharides, the vitamin C content decreased by 15%–25%. Interestingly, the presence of prebiotics (inulin and gluco‐oligosaccharides) mitigated the effects of processing on juice composition. This suggests that the addition of microcapsules may have a protective effect on juice composition during processing.

In other study UAE had a significant effect on the antioxidant content of berry pomace extracts (blackberry, chokeberry and raspberry). The quality of an extract depends on the amplitude of the ultrasound applied and the extraction time. Higher ultrasound amplitude increased TPC and antioxidant capacity (measured by ABTS—2′‐azino‐di‐(3‐ethylbenzothiazoline)‐6‐sulfonic acid) in all samples, although too long an extraction time could lead to degradation of thermolabile compounds, for example polyphenols. The highest anthocyanin content was recorded in chokeberry extracts, compared to raspberry and blackberry extracts (Piasecka et al. 2024).

Also Rueangsri et al. (2025) investigated the effect of ultrasound‐assisted aqueous two‐phase extraction parameters in various temperatures (30°C, 50°C, and 70°C), time (10, 15, and 20 min), and frequency levels (12, 24, and 36 kHz) on the extract yield and its antioxidant activity. The results exhibited that total phenolic content and total flavonoid content had the highest content when using a temperature of 70°C for 20 min and the frequency level of 12 kHz (Rueangsri et al. 2025). Combining ultrasound‐assisted extraction with microwaves also significantly increased the recovery of bioactive substances (Rueangsri et al. 2026).

Further studies found that either solvent type, as well as the other parameters of the extraction process, have an impact on the level of bioactive compounds in the extracts. It has been demonstrated that the most efficient method was extraction in a water‐ethanol solvent (50/50 v/v) with hydrochloric acid, at a 1:7 material/solvent ratio, at 35°C, for 15 min, providing 93% process efficiency (Kruszewski and Boselli 2024). Kumar et al. (2021) also highlight the importance of UAE in their review. UAE can extract bioactive compounds in a very short time, at low temperature, with less energy and solvent requirements. Furthermore, Rueangsri et al. (2026) indicated that cold plasma has strong potential for widespread industrial application due to its operational simplicity and cost‐effectiveness. The latter is partly attributable to the low temperatures required by the technology, which facilitate temperature control in large processing volumes, reduce the risk of localized overheating, and consequently lower production costs.

In contrast, the industrial implementation of hybrid extraction methods is generally more challenging because it requires the integration of two distinct technologies. Although such approaches may offer advantages over standalone techniques, particularly when processing matrices that are difficult to penetrate, they typically require more sophisticated process control and extensive optimization of power–time parameters. Moreover, raw materials subjected to combined treatments may be more susceptible to degradation. These aspects were also discussed by Rueangsri et al. (2026) in the context of ultrasound–microwave‐assisted extraction.

Nevertheless, hybrid extraction methods should not be disregarded solely because of their limited scalability. They may be particularly suitable for the production of high‐value products, where the increased complexity and associated costs of the process can be economically justified.

This method better preserves the functionality of bioactive compounds. Naturally, a number of variables are important in this method, such as frequency, power, duty cycle, temperature, time, solvent type, liquid–solid ratio, which must be optimized for each plant material.

Sonication, using high‐frequency ultrasonic waves, is a modern and promising method of processing fruit juices, allowing the shelf life of products to be extended while limiting the impact on their sensory and chemical quality. This process, based on the phenomenon of acoustic cavitation, effectively inactivates micro‐organisms without the need for intensive heat treatment, thus preserving more bioactive compounds such as polyphenols, anthocyanins, and vitamin C. Studies confirm that sonication significantly increases the phenolic content and antioxidant activity of juices from various fruits such as citrus, cherries, blueberries, and strawberries. This indicates the potential of ultrasound as an alternative to traditional pasteurization methods, especially in the context of preserving the high quality and nutritional value of fruit juices. However, incorrectly selected parameters for this method or combining it with other methods, such as high temperature, can lead to greater degradation of bioactive compounds. A recently published review also explores the possibilities of combining sonication with cold plasma and pulsed electric fields. Combining these methods extends the shelf life of juices, improves sensory quality, and enhances the absorption of bioactive compounds, including phenolic compounds (Umair et al. 2025).

CAP is an emerging non‐thermal processing technology that effectively reduces microbial contamination in fruits while preserving quality attributes better than conventional heat‐based methods. It is created by exposing the process gas to a strong electric field, which leads to partial ionization of the gas. The evidence base is substantial across multiple studies. Shill and Sit (2025) report that cold plasma treatments achieve microbial log reductions of 2.0 to 5.0 depending on treatment conditions and microbial species. For fruit juices specifically, Kaur et al. (2025) found microbial inactivation ranging from 0.15 to 7.4‐log cycles, with antioxidant activity improving by up to 261% and anthocyanin levels increasing by 35%. Marasca cherry juice was subjected to CAP treatment to determine the optimal conditions for the preservation of anthocyanins and phenolic acids. The study showed that the treatment duration and sample volume had a significant effect on the content of these compounds—a shorter treatment time (3 min) and a larger volume (3 mL) allowed the preservation of a more favorable profile of anthocyanins and phenolic acids. Gas flow had no significant effect on the results (Elez Garofulić et al. 2015). The key advantage over conventional methods is that cold plasma operates at low temperatures, maintaining nutritional and sensory properties (Sanjeevagandhi et al. 2024). However, Kaur et al. (2025) note that commercial scaling remains challenging, and further industrial‐scale studies are needed to fully understand plasma‐juice component interactions.

Determining the optimal plasma processing conditions allowed this method to be compared with pasteurized and unprocessed juice samples. As a result, the use of the established plasma processing parameters maintains a more favorable anthocyanin and phenolic acid profile of the juice sample than that of pasteurized and unprocessed juice. The increase in the content of these bioactive compounds was probably due to the breakdown of fine agglomerates in the juice under the influence of plasma. In addition, a lower plasma temperature (about 50°C) than in the case of pasteurization reduced the degradation of phenols (Elez Garofulić et al. 2015). The results confirmed that plasma treatment can be an effective method for improving the bioactive content of cherry juice. Shill and Sit (2025) also emphasized that cold plasma can be regarded as a clean‐label technology due to the absence of chemical additives during processing. Furthermore, it is considered a sustainable processing method, as it is associated with reduced water and energy consumption and a lower environmental impact.

HPP is a cold pasteurization technique in which a pressure of 300 to 600 MPa is applied uniformly to a product packed in plastic bottles or packages. This method eliminates preservatives and stabilizers while effectively preserving the nutritional value, flavor, and color of fruits and vegetables (Jia et al. 2022; Pérez‐Lamela et al. 2021).

The aim of the study by Błaszczak et al. (2017) is to evaluate the effects of high hydrostatic pressure (200–600 MPa for 15 min) and storage (4°C for 80 days) on the quality of chokeberry juice. The authors analyzed the total antioxidant capacity and the content and composition of polyphenols. Fresh, untreated juice had a significantly higher total phenolic content and antioxidant capacity compared to juices treated at 200, 400, and 600 MPa for 15 min. However, the decrease in polyphenols in juices subjected to high hydrostatic pressure was not directly correlated with the increase in pressure—at 200 MPa it was 12% and at 600 MPa only 8%. It was noted that storing the juice untreated for 20, 40, 60, and 80 days reduced total antioxidant capacity and total phenolic concentration. The decrease in polyphenol content during storage was greater in untreated juice than in pressure‐treated juices (Błaszczak et al. 2017). These results indicate that the use of high hydrostatic pressure can effectively slow down the degradation of polyphenols and preserve the higher antioxidant capacity of chokeberry juice even during long‐term storage.

It is worth noting that traditional pasteurization reduces the quality of the product, so pressure treatment can help preserve the quality and antioxidant stability of the juice. This is of great importance to the juice industry and is an attractive alternative among food preservation methods (Błaszczak et al. 2017).

In another study, the use of high pressure (HHP) and heat‐assisted pressure better preserved the initial quality composition of chokeberry juice, including its color and antioxidant compound content. Heat‐assisted pressure proved more effective in extending the shelf life of the juice under refrigeration (4°C for 30 days) compared to HPP alone. Juices subjected to these methods had higher antioxidant activity and lower nutrient losses than pasteurized juices (Jia et al. 2022).

According to Yang et al. (2026) HPP effectively retains the sensory and nutritional characteristics of fruit and vegetable juices because it has minimal effect on covalent bonds and low‐molecular‐weight compounds. However, HPP alone is not sufficiently effective at inactivating endogenous enzymes associated with quality deterioration, which often makes the application of an additional mild heat treatment necessary.

PEF is a non‐thermal food processing method that involves subjecting the product to short pulses of high‐intensity electric fields, typically ranging from 10 to 60 kV/cm. This process leads to the perforation of microbial cell membranes, resulting in their inactivation without the need to elevate temperatures to those commonly used in pasteurization. This method is often selected for liquid foods, such as juices or milk (Toepfl et al. 2014). Recent research by Ozkan et al. (2025) further supports the effectiveness of PEF in fruit juice systems. The authors investigated a mixed fruit juice blend and found that PEF treatment resulted in the highest retention of bioactive compounds after in vitro digestion, including phenolics, flavonoids, and anthocyanins. Compared to thermal processing, PEF also better preserved antioxidant capacity during storage. Moreover, the advantages of PEF include relatively low energy and water consumption, making it an environmentally friendly technology (Arshad et al. 2021).

In another study, Markovinović et al. (2025) applied PEF in combination with an additional technique—high‐power ultrasound (HPU). Strawberry juice treated with the PEF + HPU combination showed a significant improvement in most of the evaluated parameters. The authors indicated that the optimal treatment times for strawberry juice were 2.19 min for PEF and 7.48 min for HPU (Markovinović et al. 2025). These findings suggest that the combination of PEF and ultrasound is an effective, non‐thermal preservation method for strawberry juice, capable of maintaining anthocyanin stability, color, and physicochemical properties during refrigerated storage. It may serve as an alternative to conventional thermal treatments in the production of functional fruit beverages. Based on the same study, conclusions can also be drawn regarding the effect of oxygen content on anthocyanin levels. The differences observed among the juice samples were primarily associated with variations in the concentration of dissolved oxygen in the material. Oxygen promotes the oxidation of bioactive compounds, such as polyphenols and anthocyanins, leading to their degradation. Consequently, both the total polyphenol content and the antioxidant activity of the juice decrease.

The study by Markovinović et al. (2025) demonstrated that the application of technologies such as PEF and HPU resulted in a reduction in dissolved oxygen content. The authors attributed this effect, in the case of PEF, to the electroporation of cell membranes, which facilitates the removal of air from fruit tissues. During ultrasonic treatment, dissolved gases form bubbles that rise to the surface and are subsequently removed from the juice. Furthermore, the combined application of PEF and HPU exhibited a synergistic effect, with the lowest concentration of dissolved oxygen observed at the longest treatment duration (PEF 4.5 min + HPU 7.5 min).

However, it should be noted that the effect of preservation methods on polyphenol content and antioxidant activity may be twofold. On the one hand, oxygen reduction protects phenolic compounds from oxidation; on the other hand, intensive technological treatments may promote the release of polyphenols from plant cells or lead to their partial degradation. Therefore, individual samples may exhibit different levels of antioxidant activity and polyphenol content depending on the type of technology applied and the duration of treatment.

In orange juice, non‐thermal processing methods such as PEF and high hydrostatic pressure (HHP) resulted in less vitamin C loss during refrigerated storage compared to pasteurization (Esteve and Frigola 2008).

High pressure homogenization (HPH) is a non‐thermal liquid food processing technology in which the fluid is forced under high pressure (up to 400 MPa) through a homogenizing valve. This process leads to particle size reduction, microbial inactivation, and modification of food constituents. HPH is applied in various sectors of the food industry, including fruit juice stabilization and probiotic food development (Patrignani and Lanciotti 2016).

Cloudy blackcurrant juice was subjected to HPH. The highest quality retention was achieved with a single pass, an inlet temperature of 4°C, and any of the tested pressures (50–220 MPa), as well as with 20°C and 150 MPa. Deviations from these parameters resulted in increased antioxidant capacity of the juice but also led to greater losses of vitamin C and anthocyanins. It could be explained by the enhanced release of bound phenolic compounds from the plant matrix and improved extractability of intracellular antioxidants under higher mechanical stress. However, these conditions could also lead to greater losses of vitamin C and anthocyanins, which are more susceptible to temperature‐induced degradation. Moreover, an increase in pressure may enhance the aggregation of juice components, leading to a reduction in the amount of detectable dissolved substances. This can be indirectly observed through the clarification of the juice under high‐pressure treatment; however, compared to pasteurization, HPH caused fewer visual changes in the juice, such as turbidity and color degradation (Kruszewski et al. 2021). In another study on mulberry juice, increased numbers of passes during HPH were also associated with a decline in the stability of bioactive compounds, particularly anthocyanins and phenolic acids (Yu et al. 2014). Interestingly, in pomegranate juice, HPH processing resulted in an increase in total phenolic content (Turan et al. 2024).

Juice processing companies can simultaneously improve economic and environmental sustainability while maintaining quality by adopting innovative processing technologies and implementing systematic waste valorization strategies. Multiple studies point to emerging non‐thermal technologies—including HPP, PEF, and ultrasound—that preserve bioactive compounds and extend shelf‐life while reducing energy consumption (Nonglait et al. 2022; Afraz et al. 2024; Santini and Toledo 2025). In recent literature, PEF technology emerges as the most effective energy‐saving intervention for fruit juice processing while maintaining product quality. Landi et al. (2025) demonstrated that PEF pasteurization with heat recovery achieved substantial reductions compared to conventional HTST pasteurization: 20% reduction in electricity consumption, over 60% reduction in fuel gas usage, and approximately 30% reduction in greenhouse gas emissions. Thermosonication similarly reduces processing temperature and time while maintaining nutritional and sensory properties (Kesavan et al. 2023). However, the available sources lack direct comparative studies quantifying relative effectiveness across all interventions simultaneously, limiting definitive ranking of these technologies. One case study demonstrated a mobile processing unit—a modular food processing system designed to perform processing operations at or near the source of raw materials—using spiral filtration, a gentle juice separation and extraction method based on spiral flow and selective phase separation, and PEF achieved 15% environmental impact reduction versus thermal pasteurization (Silva et al. 2024). The study results indicate that decentralized and local food processing may contribute to reducing the environmental impact of supply chains and support more sustainable food production systems.

## 3 Conclusions

Current studies show that processing methods are very important for the quality of juices and their content of bioactive compounds that affect antioxidant properties. As discussed earlier, traditional pasteurization, although effective in eliminating microorganisms, often leads to the degradation of valuable bioactive compounds. Therefore, there is growing interest in newer juice preservation methods. Currently, in addition to the mild pasteurization and microwaving, there is an increasing interest from the food industry in HPP technology. HPP eliminates the use of chemical preservatives and pasteurization, preserving the nutritional value, flavor, and color of the juices and reducing the loss of bioactive compounds. Another novel method of preservation is sonication, based on the use of ultrasonic waves, which appears to be a promising alternative to traditional pasteurization. Sonication enables effective inactivation of microorganisms at lower temperatures, especially when combined with thermal processing, which enhances the overall antimicrobial effect through synergistic action, reducing the loss of thermolabile bioactive components such as vitamin C, anthocyanins, and polyphenols. The parameters of the sonication process, such as frequency, amplitude, and duration, are crucial. Further research is needed to develop optimal sonication parameters for different types of fruit juices. Particular attention should be paid to the effects of ultrasound on sensory properties, storage stability, and interactions with bioactive compounds and prebiotics. CAP processing is also a modern method. This method, although still at a rather experimental stage, allows the loss of bioactive compounds such as anthocyanins or phenolic acids to be reduced. Both methods, plasma and high pressure, offer interesting possibilities for the juice industry, enabling the production of high‐quality products, rich in bioactive compounds and with an extended shelf life. However, further research is still needed to optimize the process parameters and their impact on the content of bioactive compounds.

## Author Contributions

**Julia Soja:** conceptualization, formal analysis, writing – original draft, writing – review and editing, methodology. **Dariusz Nowak:** conceptualization, formal analysis, writing – original draft, writing – review and editing, methodology.

## Funding

The authors have nothing to report.

## Ethics Statement

The authors have nothing to report.

## Conflicts of Interest

The authors declare no conflicts of interest.

## Data Availability

The authors have nothing to report.
